# Reversibility of Defective Hematopoiesis Caused by Telomere Shortening in Telomerase Knockout Mice

**DOI:** 10.1371/journal.pone.0131722

**Published:** 2015-07-02

**Authors:** Aparna Raval, Gregory K. Behbehani, Le Xuan Truong Nguyen, Daniel Thomas, Brenda Kusler, Alina Garbuzov, John Ramunas, Colin Holbrook, Christopher Y. Park, Helen Blau, Garry P. Nolan, Steven E. Artandi, Beverly S. Mitchell

**Affiliations:** 1 Stanford Cancer Institute and Division of Hematology, Department of Medicine, Stanford University, Stanford, CA, 94305, United States of America; 2 Baxter Laboratory for Stem Cell Biology, Department of Microbiology and Immunology Stanford University, School of Medicine, Stanford, CA, 94305, United States of America; 3 Institute for Stem Cell Biology and Regenerative Medicine, Stanford University, Stanford, CA, 94035, United States of America; 4 Department of Genetics, Stanford University, Stanford, CA, 94305, United States of America; 5 Human Oncology and Pathogenesis Program and Department of Pathology, Memorial Sloan-Kettering Cancer Center, New York, NY, 10065, United States of America; 6 Departments of Medicine and Biochemistry, Stanford University, Stanford, CA, 94305, United States of America; University of Newcastle, UNITED KINGDOM

## Abstract

Telomere shortening is common in bone marrow failure syndromes such as dyskeratosis congenita (DC), aplastic anemia (AA) and myelodysplastic syndromes (MDS). However, improved knowledge of the lineage-specific consequences of telomere erosion and restoration of telomere length in hematopoietic progenitors is required to advance therapeutic approaches. We have employed a reversible murine model of telomerase deficiency to compare the dependence of erythroid and myeloid lineage differentiation on telomerase activity. Fifth generation *Tert*-/- (G5 *Tert*-/-) mice with shortened telomeres have significant anemia, decreased erythroblasts and reduced hematopoietic stem cell (HSC) populations associated with neutrophilia and increased myelopoiesis. Intracellular multiparameter analysis by mass cytometry showed significantly reduced cell proliferation and increased sensitivity to activation of DNA damage checkpoints in erythroid progenitors and in erythroid-biased CD150hi HSC, but not in myeloid progenitors. Strikingly, Cre-inducible reactivation of telomerase activity restored hematopoietic stem and progenitor cell (HSPC) proliferation, normalized the DNA damage response, and improved red cell production and hemoglobin levels. These data establish a direct link between the loss of TERT activity, telomere shortening and defective erythropoiesis and suggest that novel strategies to restore telomerase function may have an important role in the treatment of the resulting anemia.

## Introduction

Telomeres are composed of repetitive TTAGGG sequences located at the ends of chromosomes [1, 2] and are essential for the preservation of genome integrity. Cell proliferation in the presence of very short telomeres can result in genomic instability, end-to-end chromosome fusions, translocations and aneuploidy [3, 4]. In addition, critically shortened telomeres induce a DNA damage response (DDR) via the p53 pathway that may result in either senescence or apoptosis [5, 6].

Telomere erosion is prevented by the activity of the telomerase ribonucleoprotein complex consisting of the reverse transcriptase (TERT), the RNA template (TERC), and the dyskerin (DKC1) protein complex [6–9]. Telomerase is expressed in embryonic stem cells and in highly proliferative cells like HSPC to prevent replicative senescence but is absent in most somatic cells, leading to progressive telomere shortening during cell division [10, 11]. A majority of cancer cells over-express telomerase, resulting in an “immortal” phenotype [12].

Late generation telomerase-deficient mice, *Tert*-/- and *Terc*-/-, have been useful models for studying consequences of telomere dysfunction on HSC function, proliferative potential, and replicative lifespan during serial transplantation [11, 13–16]. The extended replicative capacity conferred by telomerase on HSC is essential to maintain blood cell numbers throughout the life span of an organism, while telomere shortening as a function of age contributes to the accumulation of damaged DNA and diminished replication capabilities [3, 17]. Of note, both aging and telomere shortening not only compromise HSC replication but have differential effects on lineage commitment. Telomere dysfunction impairs B lymphopoiesis but increases myeloid cell numbers in aging *Terc*-/- mice [15, 18]. This finding is consistent with reports showing that aging biases HSC differentiation towards the myeloid lineage at the expense of the lymphoid lineage and results in up-regulation of genes associated with myeloid differentiation as well as an increased incidence of myeloid malignancies [19–21]. However, the impact of telomere shortening on the commitment of HSC to erythroid differentiation has not been delineated. Clinically, telomerase gene mutations define the human bone marrow failure syndrome DC and have been identified in 15% of patients with AA and a small number of MDS patients [22–29]. In addition, shortened telomeres are present in subsets of DC, AA and MDS patients in the absence of defined mutations [30–33]. As these patients most often present with anemia, it suggests that telomerase activity and telomere length are important determinants of erythropoietic potential.

To better understand the relationship between telomerase activity and erythropoiesis, we employed a *Tert* knockout mouse model that contains a Cre-inducible Lox-Stop-Lox cassette, enabling reconstitution of telomerase activity after tamoxifen treatment. Detailed analysis of bone marrow HSPC using single cell flow and mass cytometry demonstrated differential effects of loss of telomerase activity on erythroid versus myeloid progenitors that was reversible with restoration of telomerase activity. These findings demonstrate significant impairment of erythropoiesis due to selective susceptibility of erythroid progenitors to cell cycle arrest due to DNA damage induced by shortened telomeres.

## Methods

### Animals

All animals were maintained and utilized in compliance with Stanford University’s IACUC regulations. At Stanford, the IACUC is known as Administrative Panel on Laboratory Animal Care (APLAC) that approved this study (IRB protocol # 10685).

### TRAP Assay

TRAP was performed using gel-based telomerase detection kit (S7700, Millipore) for mouse samples following the manufacturer’s instructions. Protein lysates were made from ES cell colonies or ileum from different mice using Chaps buffer. A fluorometric detection kit (S7707, Millipore) was used for TERT activity measurement in sorted human RFP+ cells according to manufacturer’s instructions. Fluorescently labeled TRAP products were quantitated using spectrofluorometer and the results converted into telomerase activity using a standard curve generated by control template (TSR8).

### Quantitative PCR Measurements of Telomere Length

DNA was extracted from c-Kit-enriched BM cells and mean telomere length was analyzed using a quantitative PCR assay as described [34]. All samples were analyzed in triplicate using an ABI 7900HT thermal cycler (Applied Biosystems).

### Interphase Fluorescence *In Situ* Hybridization Assay

To measure telomere length in rare hematopoietic progenitor populations, Q-FISH was performed as described [35]. Sorted MEP and GMP populations were cytospun onto slides using a cytocentrifuge (500rpm for 5 min) and fixed for 10 min in 4% paraformaldehyde at RT. Fixed cells were washed extensively in PBS, dehydrated consecutively in 70%, 95% and 100% ethanol for 5 min each and allowed to dry completely. Hybridization was performed in 70% formamide, 1 mg/ml blocking reagent (Roche), 10 mM Tris-HCl pH 7.2, containing PNA probe Cy3-OO-(CCCTAA)3 (Biosynthesis Inc.) by denaturing cells at 80°C for 6 min followed by O/N incubation at 4°C in the dark. Cells were then washed twice with 70% formamide, 10 mM Tris-HCl pH 7.2 and twice in PBS. DNA was counterstained with DAPI and slides were mounted in antifade reagent (ProLong Gold, Invitrogen). Digital 2D pictures were captured using a phase contrast microscope (Nikon Eclipse Ti-S) for telomere Q-FISH.

### Measurement of Telomere Length by Q-FISH

Measurement of telomere length from 2D pictures of sorted cells after FISH assay (15–20 tiff files/samples) was performed using the Telometer software (http://demarzolab.pathology.jhmi.edu/telometer/) as published in [35]. The algorithm performs subtraction of background noise, distinction of the individual telomere spots, removal of halos and separation of conjoined telomeres. The program generates statistics over the entire nucleus, as well over individual telomere in the nucleus. Statistics returned include the intensity sum of all Cy3 telomere pixels for a given nucleus that are proportional to telomere length.

### Telomerase Reactivation in G5 *Tert*-/- Mice

LSL construct was deleted by treating animals with tamoxifen (diluted in sunflower oil; Sigma-Aldrich). 8–11 months old animals were treated with either tamoxifen (1 mg) or sunflower oil (vehicle) every 3 days for a total of 5 treatments over 15 days, and allowed to recuperate for 3 months. CreER-induced excision of the LSL construct was confirmed by PCR using primers (*Tert* in2F/R) flanking the BsiWI site where the LSL cassette was inserted. PCR amplification confirms cre excision, primers used are listed in S1 Table.

### Cell Staining and Flow Cytometry

Single cell suspensions of mouse BM cells were prepared and the staining for HSC and progenitor cells was performed as described previously [36]. The immunophenotype of the populations studied is listed in the Panel A in S1 Fig

### Mass Cytometry

Mass cytometry staining and measurement was performed as previously described [37–39]. Briefly, single cell suspension of murine BM samples were prepared and were incubated in 10uM 5-iodo-2-deoxyuridine (IdU; Sigma-Aldrich) for 15 min at 37°C followed by fixing using a protein stabilization buffer (SmartTube) for 10 min at RT and the aliquots were stored at -80°C.

Bone marrow cells from the femurs of different mice were incubated in serum-free RPMI media supplemented with 10uM 5-iodo-2-deoxyuridine (IdU; Sigma-Aldrich) for 15 min at 37°C. Cells were then immediately fixed using a protein stabilization buffer (SmartTube) for 10 min at room temperature (RT) and the aliquots were stored at -80°C. Prior to mass cytometry analysis, cells were rapidly thawed in a 4°C water bath, and washed twice with cell staining media (CSM; 1xPBS with 0.5% bovine serum albumin and 0.02% sodium azide). Metal-tag barcoding was then performed as described in [37, 40] to allow all the 16 samples to be stained simultaneously in a single staining mixture.

Following barcoding, anti-CD16/32 antibody was added to the combined cell pellet for 5 min (to block Fc receptors) followed by the addition of the remainder of the surface marker-staining cocktail. Approximately 4 million BM cells of each sample were stained with a single cocktail of 3.2 mL separately for both the surface and intracellular cocktails. Surface marker staining was performed for 60 min at RT with continuous shaking. Cells were then fixed with 1.5% paraformaldehyde for 10 min at RT, permeabilized with 100% methanol for 10 min on ice, and washed. Intracellular staining was then carried out for 60 min at RT with continuous shaking. A complete list of the mass cytometry antibodies and staining concentrations is shown in S2 Table. All the antibodies were custom conjugated using MaxPar X8 metal chelating polymer (DVS Sciences) in accordance with the manufacturer’s instructions.

Following the intracellular staining, cells were washed twice with CSM and incubated overnight in PBS with 1:5000 dilution of the iridium intercalator pentamethylcyclopentadienyl-Ir(III)-dipyridophenazine (DVS Sciences, Toronto, Canada) and 1.6% paraformaldehyde (to fix antibodies to cellular antigens). Excess intercalator was removed with CSM wash followed by two washes in distilled-deionized water. Cells were re-suspended in distilled-deionized water at approximately 1 million cells/mL. Cell events were acquired on the CyTO mass cytometer (DVS Sciences) at an event rate of 150–300 events per second with internally calibrated dual count detection [41, 42]. Noise reduction and cell extraction parameters were as follows: cell length 10–75, lower convolution threshold 100 (intensity). A cell subtraction value was set to -100. After acquisition, the effect of the cell subtraction setting was negated by subtracting a value of 100 from every channel of each FCS file using the FlowCore package for R (10). The above manipulations allow for the estimation of mass signals below background and a more accurate representation of experimental noise. Normalization of signal intensity loss during the course of the 13-hour run was performed as described before utilizing metal standard beads mixed with the sample during the data acquisition [43]. Deconvolution of the barcode was performed as previously described [37].

### Statistical Analysis

ANOVA methods were used to analyze variables of interest between relevant groups of mice (WT, G0, G1, G5 and Tx G5), where WT denotes mice with the wild type allele, G0 denotes heterozygous TERT mice, G1 denotes first generation TERT-deficient mice, G5 denotes fifth generation TERT-deficient mice and TxG5 denotes tamoxifen-treated fifth generation TERT-deficient mice where TERT expression is restored. Variables of interest included telomere length, body weight, complete blood counts (CBCs), percentage of cell populations in the bone-marrow as listed in Panel A in S1 Fig, DNA damage response and cell proliferation.

Unless otherwise stated, p-values comparing two means were calculated using the two-tailed unpaired Student’s *t*-test. Statistically significant differences between WT and G5 *Tert*-/- mice are indicated by asterisks in figs showing mass cytometry data (* p value < 0.05 and ** p value < 0.01). Two-way ANOVA was used to compare differences in the means of three populations of HSCs (CD150hi, CD150lo and CD150 negative) among mice genotypes. The statistical analysis was performed in Prism version 6 (GraphPad Software, Inc. La Jolla, CA). A p-value of less than 0.05 was considered statistically significant.

## Results

### Telomere Shortening in Late Generation Telomerase Knockout Mice

**Fig 1:**
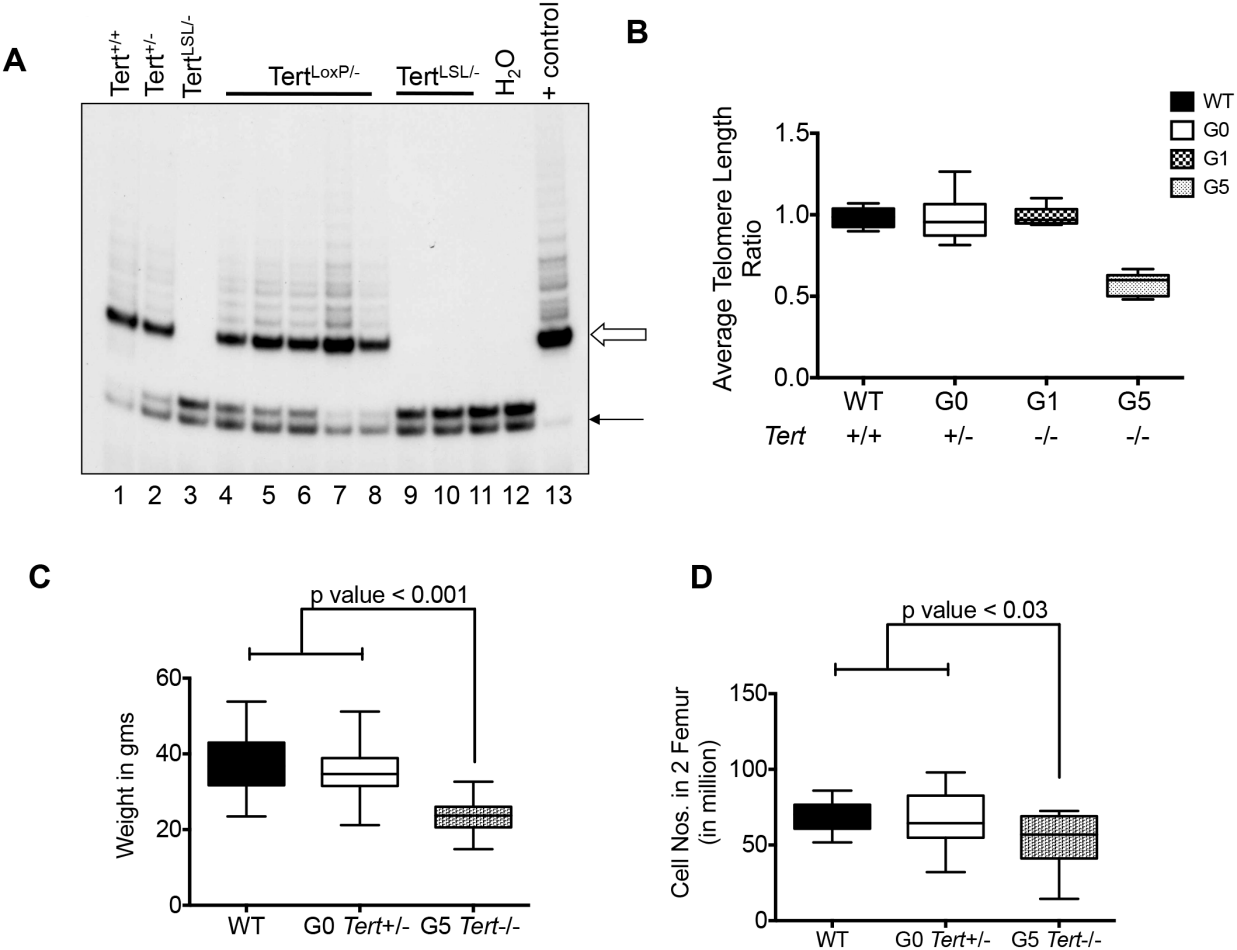
Generation and Characterization of *Tert*-/- Mice. (A) Lanes 1–3: TRAP assay for telomerase enzymatic activity using 1μg protein extracts from *Tert*+/+, *Tert*+/- and *Tert-*/- (*Tert* ^LSL/-^) embryonic stem (ES) cells; Lanes 4–8: *Tert* ^LSL/-^ clones transfected with a CMV-cre plasmid that deletes the LSL cassette; Lanes 9–11: *Tert* ^LSL/-^ clones that retained the LSL cassette; Lanes 12–13: negative (buffer only) and positive controls (293T cells), respectively. Open arrow indicates telomerase-mediated addition products and black arrow indicates internal PCR control. (B) Whisker plot of relative telomere length ratios as determined by PCR in cKit+ cells. Average telomere length was calculated as the ratio (T/S) of the telomere repeat copy number (T) to that of a single copy gene, 36B4 (S). (C) Body weights of WT (n = 15), G0 *Tert*+/- (n = 29) and G5 *Tert*-/- (n = 13) mice, aged 11–20 months. (D) Total cell numbers isolated from two femurs of WT (n = 8), G0 *Tert*+/- (n = 19) and G5 *Tert*-/- (n = 13) mice. The ends of the whiskers represent minimum and maximum values while the bar indicates the median value (50^th^ percentile). p values are based on a 2-tailed *t* test.

Heterozygous and homozygous TERT-deficient mice (G0 *Tert*+/- and G1 *Tert-*/-) were generated as described in supplemental methods and the genotype was confirmed by PCR analysis (Panel B and C in S1 Fig). A TRAP assay showed reduced telomerase activity in G0 *Tert*+/- ES cells compared to WT and undetectable activity in *Tert*-/- (*Tert* ^LSL/-^) ES cells (Fig 1A). First generation (G1) *Tert*-/- mice had telomere length equivalent to those in wild type mice, while G5 *Tert*-/- mice had significant reductions in telomere length (Fig 1B). G5 *Tert-/-* mice were also smaller with reduced numbers of cells in the BM (Fig 1C and 1D). Peripheral blood counts obtained from G5 *Tert-/-* mice aged 10–16 months revealed that the G5 *Tert-/-* mice were anemic compared to G0 *Tert*+/- mice (Table 1) with significant reductions in RBC numbers and hemoglobin and a modest increase in MCV and platelet numbers. Although total WBC numbers did not differ significantly between G0 *Tert*+/- and G5 *Tert-/-* mice, there was an increase in the relative numbers of neutrophils and a decrease in lymphocytes. Metaphase analysis of G5 *Tert*-/- spleen cells also showed reduced telomeres (Panel D in S1 Fig). G5 *Tert-/-* spleens were enlarged (data not shown), with an increase in erythroblasts and HSCs consistent with the presence of extramedullary hematopoiesis that is commonly observed in mice with defects in RBC production (Panel A and B in S2 Fig) [44]. Hematoxylin/eosin stained sections of the BM demonstrated erythroid hypoplasia with normal granulocytic maturation (Panel C and D in S2 Fig) and dysplasia was not evident (data not shown).

**Table 1:** Peripheral blood counts from G0 *Tert*+/- and G5 *Tert*-/- mice.

	G0 *Tert*+/- (n = 7)	G5 *Tert*-/- (n = 24)	p value
RBC (M/ul)	9.90 ± 0.13	8.41 ± 0.22	0.001
HGB (gm/dL)	14.79 ± 0.26	13.00 ± 0.38	0.023
HCV (%)	42.81 ± 0.71	38.28 ± 0.91	0.015
MCV (fL)	43.57 ± 0.58	45.66 ± 0.49	0.039
MCH (pg)	14.64 ± 0.46	15.28 ± 0.16	0.116
MCHC (g/dL)	34.27 ± 0.27	33.46 ± 0.15	0.020
Platelets (K/ul)	1065 ± 76.60	1324 ± 70.31	0.040
WBC (K/ul)	4.40 ± 0.66	5.05 ± 0.46	0.492
Neutrophils (%)	21.14 ± 1.62	31.17 ± 2.42	0.039
Lymphocytes (%)	71.14 ± 2.45	60.83 ± 2.86	0.0716

p values are calculated using two-tailed unpaired Student’s *t*-test.

### Reduced Erythroid Progenitors in G5 *Tert-/-* Bone Marrow

**Fig 2:**
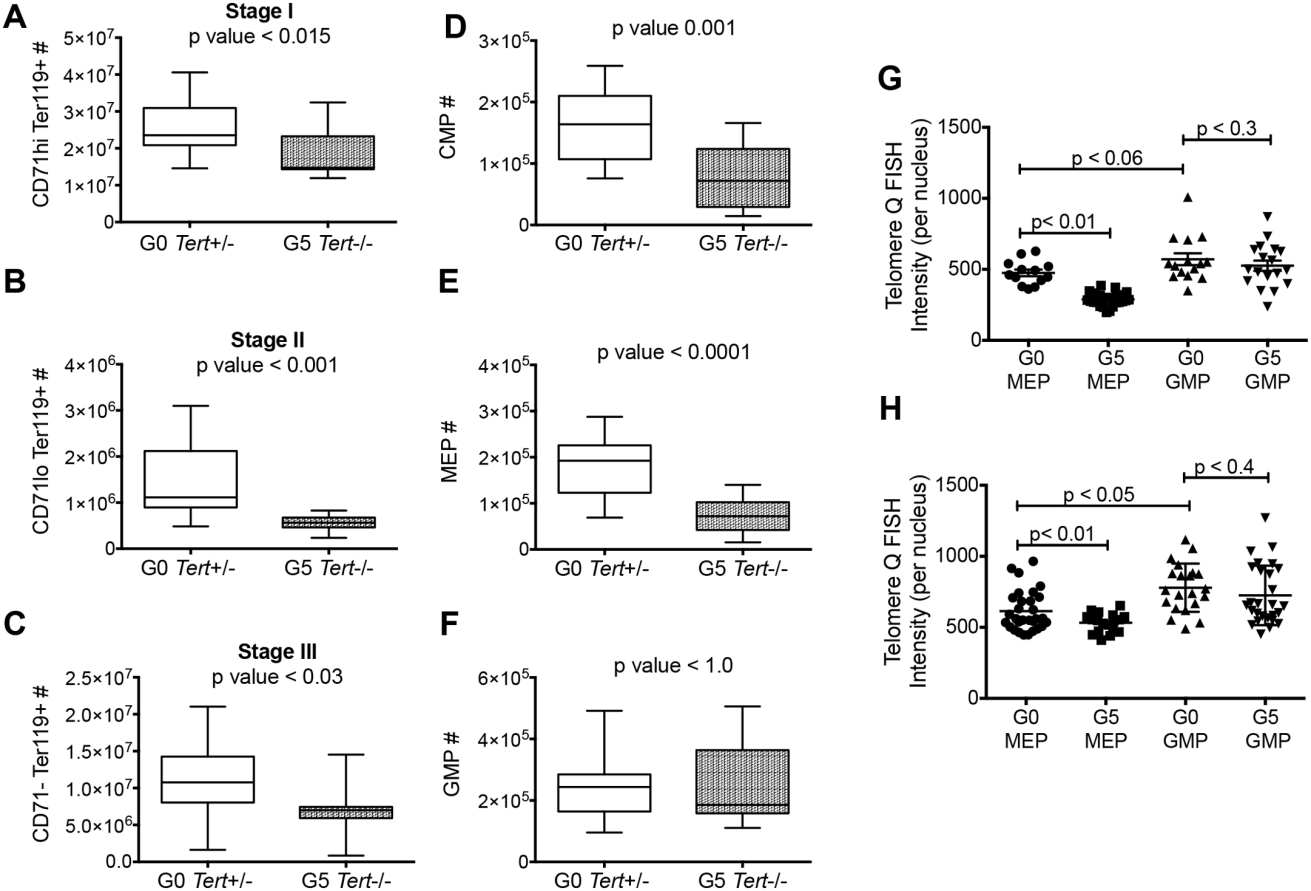
Defective Erythropoiesis in G5 *Tert*-/- mice. (A-C) Murine BM cells were separated based on levels of CD71 and Ter119 expression into three stages (I-III) of erythroid cell maturation. Whisker plot represents the number of (A) CD71hi; (B) CD71lo; (C) CD71- erythroblasts in the femur of G0 *Tert*+/- (n = 17) and G5 *Tert*-/- (n = 11) mice aged 11–20 months. (D-F) CD34 and FcγRII/III surface expression in lineage negative BM cells in G0 *Tert*+/- and G5 *Tert*-/- mice to define CMP (Lin-c-Kit+ Sca1- CD34+ FcγRII/IIIlo), MEP (Lin-c-Kit+ Sca1- CD34- FcγRII/III-) and GMP (Lin-c-Kit+ Sca1- CD34+ FcγRII/IIIhi) populations. Whisker plots represent the number of (D) CMP, (E) MEP and (F) GMP in G0 *Tert*+/- (n = 17) and G5 *Tert*-/- (n = 11) mice aged 11–20 months. The ends of the whiskers represent minimum and maximum values and the bar indicates the median value (50^th^ percentile). (G and H) Q-FISH assay showing telomere length from 15–30 interphase nuclei comparing sorted MEP and GMP populations from G0 *Tert*+/- and G5 *Tert*-/- mice using Telometer software. Data shown is from two of the three experiments.

Flow cytometric analysis of erythroblasts showed that BM from G5 *Tert-/-* mice had significantly reduced absolute numbers of erythroblasts at all stages of differentiation (Stage I, CD71hiTer119+; Stage II; CD71loTer119+; Stage III; CD71-Ter119+) (Fig 2A–2C and Panel A in S3 Fig,) as compared to G0 *Tert*+/- mice. A similar decrease in their percentage was present in G5 *Tert*-/- mice (Panel B–D in S3 Fig). The decrease in both the absolute numbers as well as in the percentage of cells confirms that the decrease in erythroblasts is not due to small size of the bones in G5 *Tert*-/- mice. An increase in the number of myeloid cells (Gr-1+Mac1+) and a decrease in the number of B cells (B220+) were also present, as has been reported previously for *Terc*-/- mice [15].

We next separated lineage-, c-Kit+ and Sca1- cells based on CD34 and FcγRII/III expression to determine changes in committed erythroid and myeloid progenitors in the BM (Panel E in S3 Fig). Both common myeloid progenitors (CMP; Lin-c-Kit+ Sca1- CD34+ FcγRII/IIIlo) and megakaryocyte-erythroid progenitors (MEP; Lin-c-Kit+ Sca1- CD34- FcγRII/III-) numbers were significantly reduced in the G5 *Tert-/-* mice, while granulocyte-macrophage progenitor (GMP; Lin-c-Kit+ Sca1- CD34+ FcγRII/IIIhi) numbers were unaffected (Fig 2D–2F). The percentage of CMP and MEP, but not GMP, was also decreased among cKit+ progenitor cells (Panel F–H in S3 Fig). However, the number of T cells (CD3+) and megakaryocytes (CD41+) did not differ (Panel A–C in S4 Fig and data not shown).

To examine the functional capabilities of progenitor populations, we compared the colony forming ability of total BM cells from G0 *Tert*+/- and G5 *Tert-/-* mice. There was a significant increase in CFU-GM (colony forming unit-granulocyte/monocyte) colony number in the G5 *Tert-/-* mice, while the numbers of CFU-GEMM (colony forming unit-granulocyte/erythroid/macrophage/ megakaryocyte) colonies were significantly decreased. In addition, the total number of cells harvested on day 15 was reduced compared to controls, suggesting a reduction in cellular proliferation (Panel D and E in S4 Fig). Plating bone-marrow cells in methylcellulose supplemented with erythropoietin showed that G5 *Tert-/-* BM cells formed significantly lower numbers of CFU-E and BFU-E colonies, confirming the defect in the erythroid progenitor compartment (Panel F and G in S4 Fig).

### Reduced Telomere Length in G5 *Tert*-/- MEP Population

MEPs and GMPs from age matched G0 *Tert*+/- (n = 3) and G5 *Tert*-/- (n = 3) mice were sorted and quantitative telomere-fluorescence *in situ* hybridization (Q-FISH) assay was performed to measure telomere length. Fig 2G and 2H shows Q-FISH data from two of the three G0 and G5 pairs studied. MEPs have shorter telomeres than GMPs in both control and mutant mice, while the variation in telomere length is more pronounced in GMPs than MEPs. Importantly, there was a significant decrease in telomere length in G5 *Tert*-/- MEPs as compared to G0 *Tert*+/- MEPs that was not present in G5 *Tert*-/- GMPs.

### Altered Hematopoietic Stem Cell Numbers in G5 *Tert-/-* Mice

**Fig 3:**
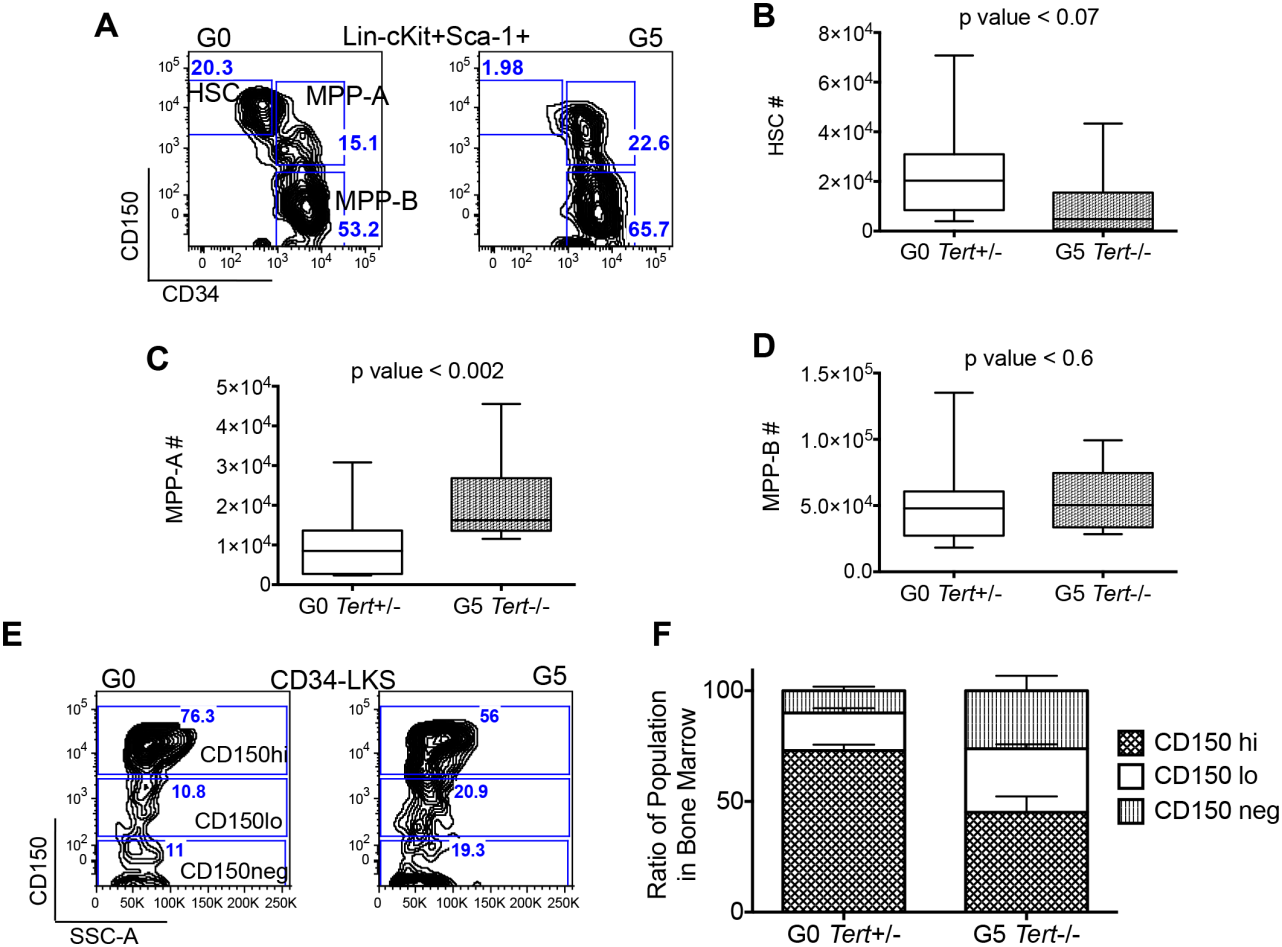
Comparison of HSC and MPP numbers between G0 *Tert*+/- and G5 *Tert*-/- mice. (A) Representative FACS profiles of lineage-, c-Kit+ and Sca1+ cells separated based on CD34 and CD150 expression, showing absolute numbers of HSC (Lin-c-Kit+Sca1+CD34-CD150+), MPP-A (Lin-c-Kit+Sca1+CD34+CD150+) and MPP-B (Lin-c-Kit+Sca1+CD34+CD150-) populations in G0 *Tert*+/- and G5 *Tert*-/- BM. (B-D) Comparison of absolute numbers of (B) HSC, (C) MPP-A and (D) MPP-B in the femurs of G0 *Tert*+/- (n = 17) and G5 *Tert*-/- (n = 11) mice aged 11–20 months. The ends of the whiskers represent minimum and maximum values while the bar indicates the median value (50^th^ percentile). p values are based on a 2-tailed *t* test. (E) Representative FACS profile of CD34-LKS cells subdivided into CD150hi, CD150lo and CD150negative fractions in G0 *Tert*+/- and G5 *Tert*-/- BM cells. (F) Ratios of the three CD150 fractions within CD34-LKS cells from G0 *Tert*+/- (n = 6) and G5 *Tert*-/- (n = 9) mice aged 11–20 months, as calculated from the absolute numbers of total HSC population. p value < 0.01(two-way ANOVA) demonstrating that the differences between the three CD150 fractions in G0 *Tert*+/- and G5 *Tert*-/- mice are statistically significant.

We then used CD150 and CD34 surface markers to differentiate HSC from MPP (multi-potent progenitor) in Lin-cKit+Sca1+ population (Fig 3A) [45]. HSC (Lin-c-Kit+Sca1+CD34-CD150+) numbers were decreased in G5 *Tert-/-* BM, while myeloid and lymphoid balanced MPP-A (Lin-c-Kit+Sca1+CD34+CD150+) numbers were significantly increased. Lymphoid-biased MPP-B (Lin-c-Kit+Sca1+CD34+CD150-) numbers were unaffected (Fig 3B–3D). Similar changes in the percentages of HSC and MPP within Lin-c-Kit+Sca1+ cells were also observed (Panel A–C in S5 Fig). Flow cytometric analysis using Flk-2 confirmed the reduction in HSC numbers (Lin-c-Kit+Sca1+CD34-Flk2-) in G5 *Tert-/-* mice (Panel D and E in S5 Fig).

Expression of CD150 in CD34-Lin-c-Kit+Sca1+ (CD34-LKS) cells divides HSCs into myeloid-biased long-term repopulating cells (CD150hi) and myeloid-lymphoid balanced HSCs with low potential for self-renewal (CD150lo) (Fig 3E) [19, 46]. Previous studies have shown that the loss of erythroid differentiation is associated with loss of CD150 expression within the CD34-LKS population [46]. In G5 *Tert*-/- mice, although CD34-LKS CD150hi numbers were reduced, the ratio between CD34-LKS CD150hi, lo and negative cells was also altered, with a higher frequency of CD150lo and CD150negative in G5 *Tert*-/- mice relative to age matched G0 *Tert*+/- mice (Fig 3F). These differences were found to be statistically significant (two way ANOVA; p value < 0.01). Thus, the significant reduction in CD150hi HSC in G5 *Tert-/-* mice could, in part, account for the decrease in erythroblasts and the anemia in these mice.

### Telomerase Reactivation Restores Normal Hematopoiesis

**Fig 4:**
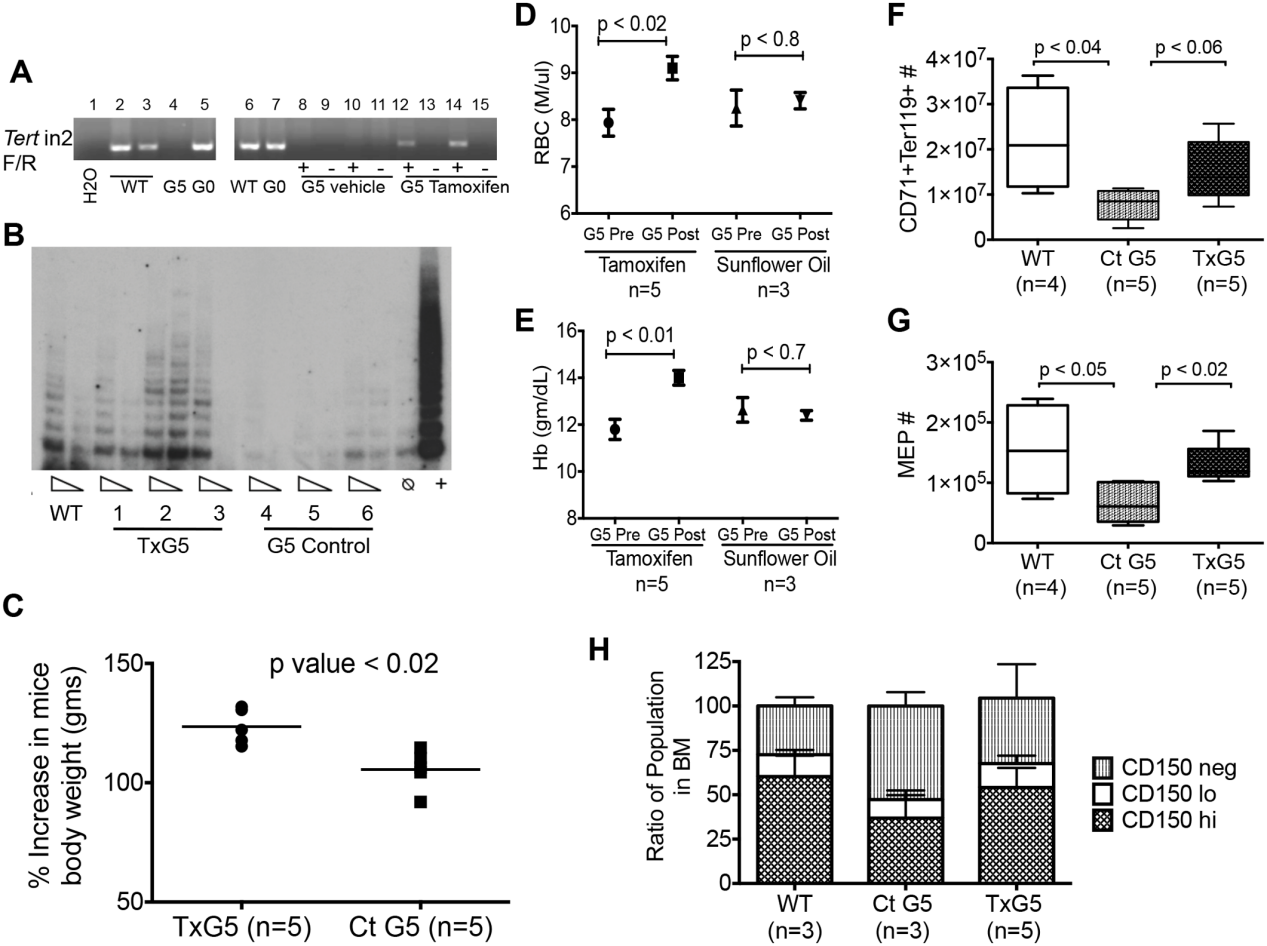
Telomerase Reactivation in G5 *Tert*-/- Mice. (A) PCR showing Cre mediated excision of LSL cassette within intron 2 (in2) of *Tert* gene using *Tert* in2 F/R primers in DNA extracted from G5 *Tert*-/- spleen: untreated (lanes 4, 9, 11, 13 and 15); vehicle treated (lanes 8 and 10); tamoxifen treated (lanes 12 and 14). WT (lanes 2, 3 and 6) and G0 *Tert*+/- spleen (lanes 5 and 7) were used as positive controls. (B) TRAP assay for telomerase activity in 1–2 μg ileum lysate from 11–14 months old WT or tamoxifen treated G5 *Tert*-/- (TxG5 Tert-/-) mice (lanes 1,2,3) or treated with vehicle (lanes 4,5,6) for 3 months. Negative (buffer only) and positive (293T lysate) controls are shown on the far right. (C) Percentage increase in body weight in tamoxifen and vehicle treated G5 *Tert*-/- mice. Bars indicate mean values. (D and E) Comparison of RBC numbers and hemoglobin levels in the peripheral blood in tamoxifen or vehicle treated G5 *Tert*-/- mice before and 3 months after treatment. (F and G) Effects of tamoxifen treatment on erythroblast (CD71+Ter119+) and MEP (Lin-c-Kit+ Sca1- CD34- FcγRII/III-) populations. Bars indicate standard deviation and p values are based on a 2-tailed *t* test. (H) Ratios of the CD34-LKS CD150 hi, CD150 lo and CD150 negative populations from WT, vehicle and tamoxifen G5 *Tert*-/- treated mice, as calculated from absolute numbers of HSC population. p value < 0.01 (two-way ANOVA). The results shown are combined data from two separate experiments.

To establish the reversibility of the effects of TERT loss on hematopoiesis, mice were treated with tamoxifen or vehicle control. LSL cassette deletion resulted in a PCR product in tamoxifen treated G5 *Tert*-/- (TxG5) mice that was absent in vehicle treated mice (Fig 4A). Tamoxifen treatment restored telomerase activity to levels similar to that in WT mice (Fig 4B) and also improved body weight, RBC counts and hemoglobin levels (Fig 4C–4E). Significant differences in RBC numbers and hemoglobin levels were not observed in control mice after tamoxifen treatment (Panel A and B in S6 Fig). Neutrophil and B lymphocyte numbers also returned to control levels in the BM by 3 months after tamoxifen treatment (Panel C and D in S6 Fig). Flow cytometric analysis of the BM from TxG5 mice revealed that the percentages of CD71+Ter119+ erythroblasts, committed erythroid progenitor cells (MEP) and CD34-LKS CD150hi, lo and negative cells were all restored to the levels found in the wild type mice (Fig 4F–4H). These data suggest that reactivation of telomerase can restore normal hematopoiesis in both stem and progenitor populations. Although a significant increase in telomere length in tamoxifen-treated G5 *Tert*-/- mice was not detected (data not shown), this is most likely attributable to the lack of sensitivity of the techniques available. However, it has been established that small increments in telomere length are sufficient to elicit cellular responses [47].

### Multiparameter Analysis by Single Cell Mass Cytometry

We used the novel technology of mass cytometry that allows for the simultaneous measurement of 19 surface markers and 13 intracellular markers (S2 Table) to further characterize changes in progenitor populations in these mice. This approach allowed us to use the additional marker, endoglin (CD105), to confirm the differences observed using standard flow cytometry with the antibody to CD34. It also enabled separation of erythroid progenitors into pre megakaryocyte-erythroid precursors (PreMegE; Lin-cKit+Sca1-FcγRII/III-CD150+CD105-), Pre-GM (Lin-cKit+Sca1-FcγRII/III-CD150-CD105-), Pre CFU-E (Lin-cKit+Sca1-FcγRII/III-CD150+CD105+) and CFU-E (Lin-cKit+Sca1-FcγRII/III-CD150-CD105+) populations [48].

Mass cytometric analyses further confirmed a significant decrease in the numbers of erythroblasts and committed erythroid progenitors (CD71+Ter119+, Pre CFU-E, CFU-E, and MEPs) in G5 *Tert-/-* mice (Panel A–D in S7 Fig). These decreases were specific to the erythroid lineage and all were corrected upon re-expression of telomerase. Myeloid progenitors (GMP and Pre GM) and the Pre MegE populations were unaffected and myeloid cell numbers (Ly-6G+Mac-1+) returned to control levels (Panel E–H in S7 Fig).

### Intracellular Signaling Analysis in HSPCs by Mass Cytometry

**Fig 5:**
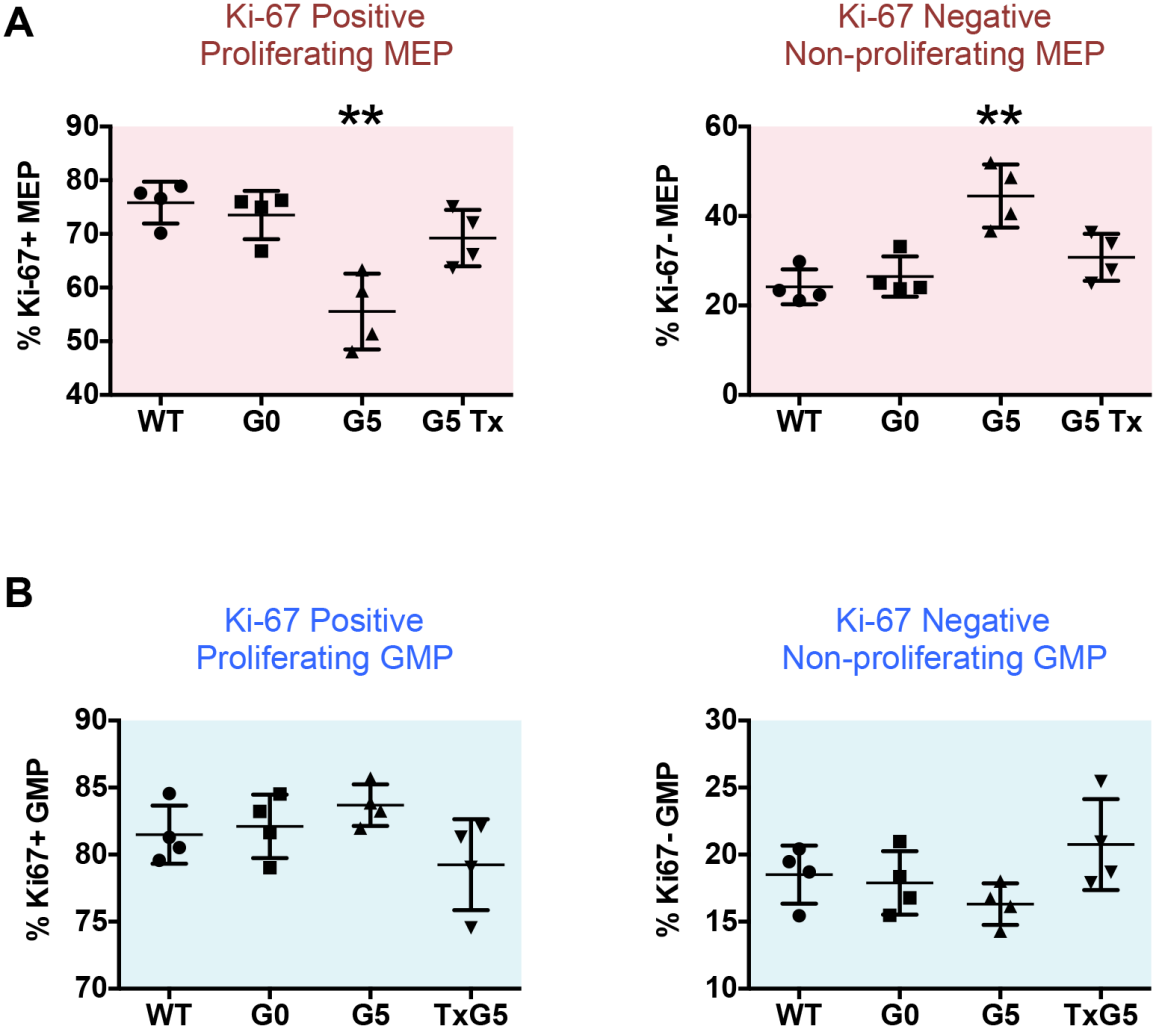
Cell Cycle Analysis of MEP and GMP Populations by Mass Cytometry. (A and B) Scatter plots comparing uridine incorporation and Ki-67 expression in MEPs and GMPs in WT, G0 *Tert*+/-, G5 *Tert*-/- and TxG5 *Tert*-/- mice. Percentages for Ki-67 + (proliferating) and Ki-67 negative (non-proliferating) cells were derived from the absolute numbers of MEPs and GMPs. Bars indicate standard deviations and the p values are based on a 2-tailed *t* test. Statistically significant differences between WT and G5 *Tert*-/- mice are indicated by ** (p value < 0.01). There were no significant differences between WT, G0 *Tert*+/- and TxG5 *Tert*-/- mice.

The number of actively proliferating MEPs in G5 *Tert*-/- mice as measured by Ki-67 was significantly reduced as was phospho Rb (pRb) expression, while the percentage of non-proliferating Ki-67 negative and pRb negative MEPs increased (Fig 5A and Panel A in S8 Fig). These effects were restored to normal levels in TxG5 mice. GMPs did not show similar changes in Ki-67 or pRb expression (Fig 5B and Panel B in S8 Fig). Ki-67 expression was also reduced in CD71+Ter119+, CFU-E and Pre CFU-E populations in G5 *Tert-/-* mice but was not affected in the Pre-GM population (Panel A–E in S9 Fig). The increase in Ki-67 expression in myeloid cells corresponded to the increase in myeloid cell numbers in these mice.

**Fig 6:**
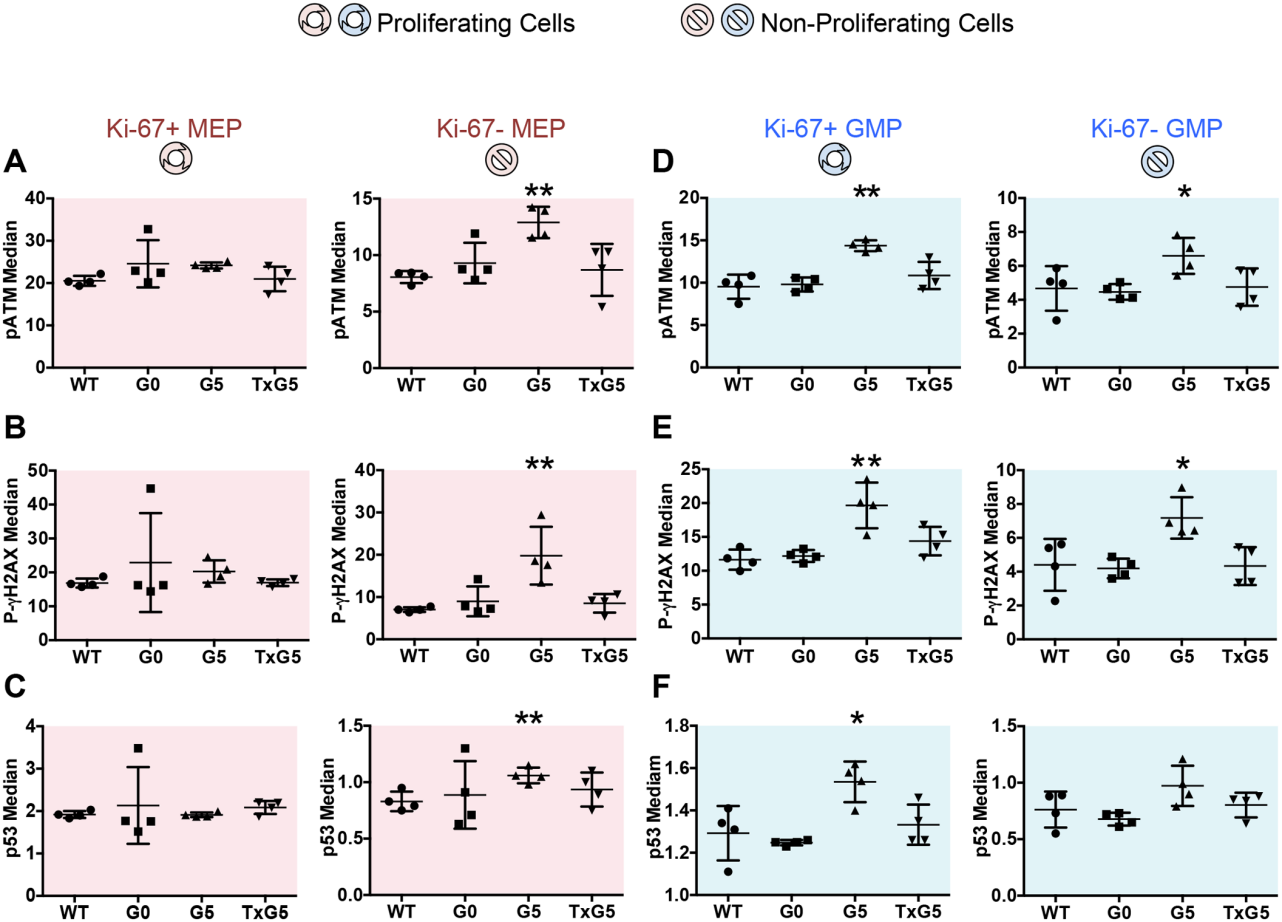
Simultaneous Determinations of Cell Cycle and DNA Damage Response in MEP and GMP Populations as Determined by Mass Cytometry. Scatter plots showing (A and D) pATM, (B and E) p-γH2AX and (C and F) p53 expression in Ki-67 positive proliferating and Ki-67 negative non-proliferating cells in MEP and GMP populations. Bars indicate standard deviations and the p values are based on a 2-tailed *t* test. Statistically significant differences between WT and G5 *Tert*-/- mice are indicated by * (p value < 0.05) or ** (p value < 0.01). There were no significant differences between WT, G0 *Tert*+/- and TxG5 *Tert*-/- mice.

MEPs and GMPs from G5 *Tert-/-* mice expressed significantly higher levels of the DNA damage markers, phosphorylated ATM (pATM) and phosphorylated γH2AX (p-γH2AX). The differences in median levels of expression were restricted to the non-proliferating cell fraction (Ki-67 negative) in MEPs (Fig 6A and 6B), but were present in both the proliferating and non-proliferating fractions in GMPs (Fig 6D and 6E). Similar differences were observed in total p53 levels in MEPs and GMPs in G5 *Tert*-/- mice, with the increase in p53 expression present only in non-proliferating MEPs (Fig 6C) but in both proliferating and non-proliferating GMPs (Fig 6F). PARP cleavage was also significantly increased in non-proliferating, but not in proliferating MEPs while caspase-3 expression was unaffected (Panel A and B in S10 Fig and data not shown). Of note, the increase in expression of markers of DNA damage returned to normal levels after restoration of telomerase activity, supporting some restoration of length in critically shortened telomeres. In sum, these data demonstrate that proliferation is significantly reduced and apoptosis is increased in MEPs in G5 *Tert*-/- mice and suggest that MEPs are more sensitive to the induction of DNA damage checkpoints resulting from telomere dysfunction. In contrast, the proliferation of GMPs is not affected despite similar increases in p-γH2AX, pATM, and p53 expression, suggesting that myeloid progenitors may be more resistant to cell cycle arrest resulting from DNA damage.

**Fig 7:**
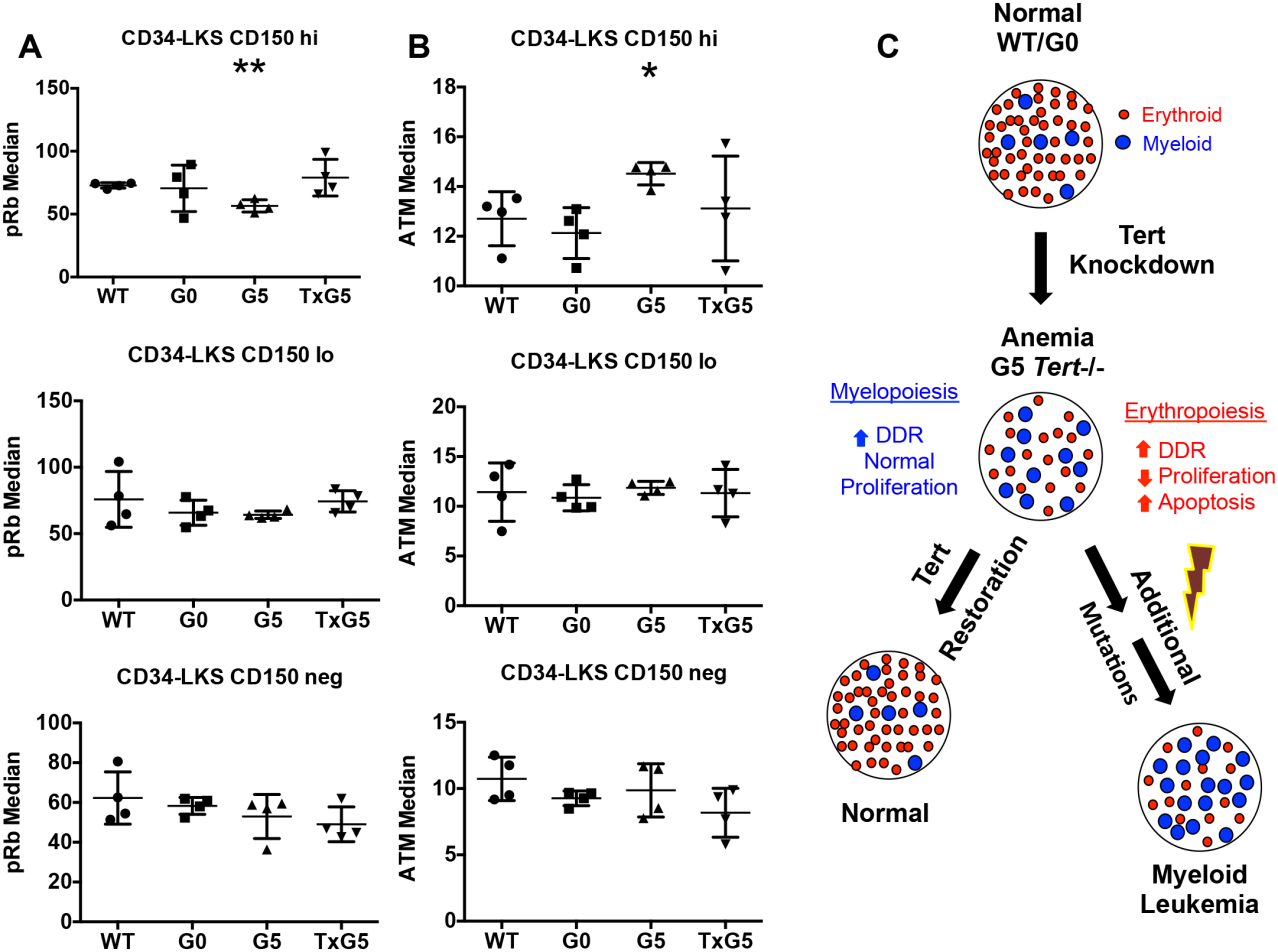
Effect of Telomerase Depletion on the Differentiation of Human CD34+ Progenitor Cells. (A-B) Scatter plots showing median values for (A) pRb and (B) pATM expression in CD34-LKS CD150hi, CD150lo and CD150neg populations from WT *Tert*+/+ (n = 4), G0 *Tert*+/- (n = 4), G5 *Tert*-/- (n = 4) and TxG5 *Tert*-/- (n = 4) mice. Bars indicate standard deviation and the p values are based on a 2-tailed *t* test. Statistically significant differences between WT and G5 Tert-/- mice are indicated by * (p value < 0.05) and ** (p value < 0.01). There were no significant differences between WT, G0 *Tert*+/- and TxG5 *Tert*-/- mice. (C) Proposed model for the consequences of reduced telomere length: erythropoiesis is decreased and myelopoiesis is increased due to differential susceptibility of erythroid and myeloid progenitors to the DDR induced by short telomeres. Reactivation of telomerase activity restores normal hematopoiesis, while cumulative DNA damage may lead to myeloid malignancies.

Similar studies carried out on HSCs demonstrated a decrease in pRb expression and an increase in pATM expression in CD34-LKS CD150hi HSCs from G5 *Tert*-/- mice (Fig 7A and 7B). This difference was not observed in CD150lo or negative HSC population. pRb expression was restored in the CD150hi population after tamoxifen treatment. Ki-67 expression was reduced and Caspase-3 expression was increased in the CD34-LKS CD150hi HSC population, although this difference was not statistically significant (data not shown).

## Discussion

Telomere shortening is common in DC, AA and MDS patients [30–33], but the specific consequences of telomere erosion for hematopoiesis have not been well-defined. Our data demonstrate that significant telomere shortening in late generation G5 *Tert*-/- mice results in impaired erythroid maturation and anemia and a significant reduction in both early and committed erythroid progenitors, as well as in the numbers of CD150hi-expressing HSCs that are associated with erythroblast differentiation. In contrast, myeloid cell numbers were increased. These differences were not present in first generation G0 *Tert*+/- or in G1 *Tert*-/- mice, suggesting that it is the telomere length and not telomerase per se that is essential for erythropoiesis. The use of mass cytometry using 32 antibodies simultaneously enabled the analysis of intracellular signaling pathways in rare HSPC populations. Results showing a significant decrease in Ki-67 and pRb expression in erythroid, but not myeloid, progenitors and in CD150hi HSCs in G5 *Tert*-/- mice indicate a selective reduction in cell proliferation. A small but significant increase in apoptosis was also noted in Ki-67 negative MEPs. Importantly, these defects were all reversed with reactivation of telomerase activity, resulting in restoration of erythroblasts and erythroid progenitors and CD150hi HSC in the BM and improved RBC numbers and hemoglobin levels in the peripheral blood. The expression of pATM and p-γH2AX in these populations also declined, demonstrating the reversibility of DNA damage. We conclude that defective hematopoiesis, and in particular erythropoiesis, resulting from loss of telomerase can be reversed with restoration of telomerase activity (Fig 7C).

An increase in telomere length upon reactivation of telomerase activity was not measurable in progenitor or lineage positive cells. This result is not unexpected as its been shown that it takes as many generations for telomeres to return to wild type levels as it takes for them to shorten through the intercrossing of heterozygous mice [49]. Hence it is possible that the 3 months of tamoxifen treatment was not of long enough duration to produce a measurable increase in telomere length. It would seem more likely, however, that the reconstitution of telomerase might be sufficient to elongate the shortest telomeres in this interval [47], thereby eliminating the DNA damage signal that mediates cell cycle arrest.

Telomere length has been shown to correspond to the expansion potential of cord blood CD34+ cells along erythroid but not myeloid or megakaryocyte lineages [50] and individuals with shortened telomeres have lower RBC counts [51]. Furthermore, *ex vivo* differentiation of human cord blood CD34+ cells into myeloid, erythroid and megakaryocytic cell populations is associated with transient up-regulation of TERT activity only in the erythroid cultures, suggesting that erythroid expansion is more dependent on TERT activity. The transcriptional up-regulation of *Tert* mRNA by erythropoietin via the JAK2/STAT5 pathway further emphasizes its importance for RBC production [52]. These studies, in conjunction with our own, support the concept that telomerase function is more important for erythropoiesis than for myelopoiesis.

One explanation for this observation is that erythroid progenitors must divide more frequently to supply the requisite numbers of mature erythroid cells. The greater shortening of telomeres in erythroid as opposed to myeloid progenitors in G5 *Tert*-/- mice supports this concept. The reduced expression of p-ATM and p-γH2AX in GMPs as compared to MEPs is also consistent. However, a second possibility is that alternative telomerase-independent mechanisms are available to sustain telomere length in myeloid, but not erythroid, progenitors.

The ability to distinguish proliferating (Ki-67 positive) from non-proliferating progenitors using mass cytometry led to the observation that the p-γH2AX and pATM markers of DNA damage were elevated in proliferating GMPs, whereas they were not elevated in proliferating MEPs. These data suggest, but do not prove, that myeloid progenitors may continue to proliferate in the face of DNA damage, perhaps due to an altered DNA damage checkpoint response. They are also consistent with previous studies [50] demonstrating that the total number of population doublings in erythroid lineage cells is much more tightly linked to telomere length than in cells of myeloid lineage. It is an intriguing possibility that the markedly increased incidence of hematopoietic malignancies within the myeloid as opposed to the erythroid lineage might result from a relative lack of response in myeloid progenitors to DNA damage (Fig 7C).

Accumulation of DNA damage also underlies a diminished long term repopulating capability of HSC stem cells with short telomeres [14, 16]. We found that CD34-LKS CD150hi HSC from G5 *Tert*-/- mice also showed evidence of a DNA damage response and had reduced proliferation. Thus, the differences in myeloid and erythroid cell numbers may also be partially explained by the reduction in CD150 hi HSC and within CD34-LKS cells in G5 *Tert-/-* mice. Previous *in vitro* colony formation studies have shown that the majority of colonies formed from CD150hi CD34-LKS cells were mixed colonies (CFU-GEMM), while very few (<10%) were formed from CD150 negative CD34-LKS cells. The opposite pertained to neutrophil/macrophage colonies that arise predominantly from CD150 negative CD34-LKS cells [46]. The significant decrease in the number of CFU-GEMM colonies and the increase in CFU-GM colonies from G5 *Tert*-/- BM cells further support this hypothesis. The susceptibility of CD150hi HSC compared to the CD150lo or CD150negative HSC to telomere erosion and consequent DNA damage is somewhat surprising, as CD150 hi cells proliferate at lower rates [53]. Recently, a mechanistic link was identified between the DNA damage response in HSC and their ability to differentiate. The transcription factor Batf is up-regulated in HSC in response to DNA damage in G3 *Terc*-/- mice, promoting lymphoid differentiation [54]. It is therefore possible that another as yet unidentified transcription factor could play a role in reducing erythroid differentiation in response to loss of TERT activity.

Recently, telomere dysfunction has been linked with mitochondrial defects as it results in repression of PGC-1 genes and an associated decline in mitochondrial biogenesis in tissues including CD34loLKS cells [55]. Although PGC-1 has been shown to regulate erythropoiesis [56], we did not observe differences in its expression in sorted erythroblasts from G0 *Tert*+/- and G5 *Tert*-/- mice (data not shown).

Both the G5 *Tert*-/- mice and the control mice (WT *Tert*+/+ and G0 *Tert*+/-) were of mixed background. However, to control for strain-specific effects and to avoid a “founder” effect, G5 *Tert*-/- mice as well as the control mice (WT *Tert*+/+ and G0 *Tert*+/-) were generated through five generations of breeding of multiple pairs to produce age-matched controls. The absence of erythropoietic defects in any of the control mice strongly suggests that the consistent phenotype in G5 mice is due to lack of TERT activity and shortening of telomeres rather than to any allele variability among the mice.

In summary, the use of the combined modalities of flow and mass cytometry conclusively demonstrate that telomere length is highly relevant to erythroid maturation. They suggest that proliferating erythroid progenitors are more sensitive to DNA damage checkpoints triggered by telomere shortening, while myeloid progenitors continue to proliferate. It is possible that anemia and myeloid malignancies associated with aging [3, 57, 58] and BMF syndromes [27, 30, 59] may result in part from telomere shortening. The observation that re-activation of telomerase not only reverses anemia, but also restores homeostasis in other hematopoietic cell lineages, indicates a potential therapeutic approach, for example through *TERT* mRNA delivery [60], for bone marrow disorders that are associated with short telomeres.

## Supporting Information

S1 File Supplemental Methods. (DOCX)

S1 Fig Generation of *Tert*-/- mice, Chromosomal Abnormalities and Immunophenotypes of Cell Poplulations Studied. (DOCX)

S2 Fig Extramedullary Hematopoiesis and Histopathology of the bone marrow in G5 *Tert*-/- mice. (DOCX)

S3 Fig Defective Erythropoiesis in G5 *Tert*-/- mice. (DOCX)

S4 Fig Leukocyte cell numbers and Colony Formation Assay from G0 *Tert*+/- and G5 *Tert*-/- Bone Marrow cells. (DOCX)

S5 Fig HSC and MPP cell populations in G5 *Tert*-/- mice. (DOCX)

S6 Fig Tamoxifen Treated Control Mice and Myeloid Cell and B Cell numbers after Telomerase Reactivation. (DOCX)

S7 Fig Changes in Cell Populations as Defined By Mass Cytometry. (DOCX)

S8 Fig Cell Cycle Analysis in MEP, GMP and HSC Populations as Determined by Mass Cytometry. (DOCX)

S9 Fig Cell Cycle analysis as Determined by Mass Cytometry in Different Erythoid and Myeloid populations. (DOCX)

S10 Fig Apoptosis in MEP and GMP Populations as Determined by Mass Cytometry. (DOCX)

S1 Table Sequence of primers used for genotyping mice. (DOCX)

S2 Table Antibodies used for mass cytometry analysis. (DOCX)
