# Supplementary material for: Reversibility of Defective Hematopoiesis Caused by Telomere Shortening in Telomerase Knockout Mice
Source: PLoS One. 2015 Jul 2;10(7):e0131722. doi: 10.1371/journal.pone.0131722 (PMC4489842; doi:10.1371/journal.pone.0131722)
Supplement: S1 File — (DOCX) [file pone.0131722.s001.docx]

**Supporting Information**

**Supplemental Methods:**

**Generation of Telomerase Knockout mice:** A LoxP-Stop-LoxP (LSL) cassette was knocked into the second intron of the *Tert* gene (S1A Fig.) to generate heterozygous telomerase knockouts in R26 CreER/+ mice. The G0 *Tert*^LSL/+^ R26^CreER/+^ mice (genetic background 129/SvXC57BL/6) were bred to generate G1 *Tert^LSL/LSL^R26^CreER/CreER^* mice, which were then intercrossed to generate G2, G3, G4 and G5 *Tert^LSL/LSL^R26^CreER/CreER^* (G5 *Tert*-/-) mice. Similarly, G0 *Tert*-/- mice were intercrossed to generate age matched control mice (WT *Tert*+/+ and G0 *Tert*-/-). Primers used for genotyping are listed in S1 Table. All animals were maintained and utilized in compliance with Stanford University’s IACUC regulations, called Administrative Panel on Laboratory Animal Care (APLAC) that approved this study.

**Colony forming unit assay:** Freshly isolated whole bone marrow cells were collected from G0 *Tert+/-* and G5 *Tert-/-* mice and cells were plated in methylcellulose. Fifteen thousand cells were plated onto either Methocult M3434 (STEMCELL Technologies) for CFU-GM and CFU-GEMM colonies, or M3334 for BFU-E and CFU-E colonies. Colonies in Methocult M3434 were counted on Day 13-14 and for those on M3334, CFU-E colonies were counted on Day 3 and BFU-E colonies were counted on Day 5.
