# Supplementary material for: Reversibility of Defective Hematopoiesis Caused by Telomere Shortening in Telomerase Knockout Mice
Source: PLoS One. 2015 Jul 2;10(7):e0131722. doi: 10.1371/journal.pone.0131722 (PMC4489842; doi:10.1371/journal.pone.0131722)
Supplement: S1 Fig — (DOCX) [file pone.0131722.s002.docx]

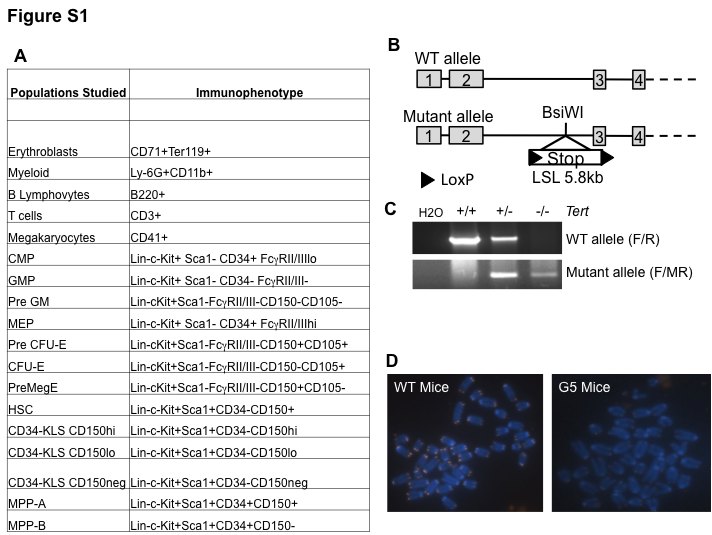


**S1 Fig. Generation of *Tert*-/- mice, Chromosomal Abnormalities and Immunophenotypes of Cell Poplulations Studied.** (A) Immunophenotypes of the hematopoietic subpopulations studied. (B) A LSL cassette was knocked into the second intron of the telomerase gene at the BsiWI site to generate telomerase heterozygous knockout mice, G0 *Tert*^+/LSL^ (G0 *Tert*+/-). (C) PCR of the WT, *Tert*+/- and *Tert*-/- mouse DNA showing heterozygous and homozygous knockout of the telomerase gene using wild type F primer with either wild type R primers or mutant R (MR) primer. (D) Metaphase of WT spleen cell showing bright telomere signal and G5 *Tert*-/- spleen cell showing weak telomere signal. In total 3 mice of each genotype was studied and ~20 metaphase chromosomes per mice were analyzed.
