# Supplementary material for: Reversibility of Defective Hematopoiesis Caused by Telomere Shortening in Telomerase Knockout Mice
Source: PLoS One. 2015 Jul 2;10(7):e0131722. doi: 10.1371/journal.pone.0131722 (PMC4489842; doi:10.1371/journal.pone.0131722)
Supplement: S2 Fig — (DOCX) [file pone.0131722.s003.docx]

**
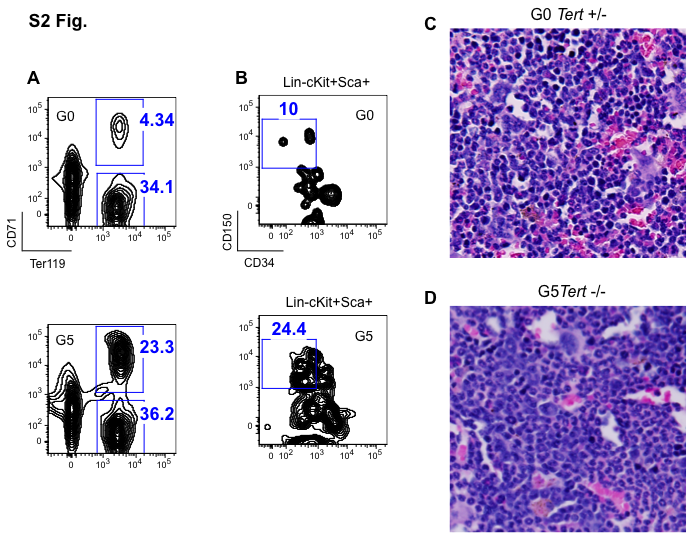
**

**S2 Fig. Extramedullary Hematopoiesis and Histopathology of the bone marrow in G5 *Tert*-/- mice.**  (A and B) Representative FACS profiles showing frequency of (A) immature erythroblasts (CD71+Ter119+) and (B) HSC (Lin-c-Kit+Sca1+CD34-CD150+) population in the spleen from G0 *Tert*+/- and G5 *Tert*-/- mice. In total 3 mice of each genotype were studied. (C and D) A representative section of bone marrow from a G0 *Tert*+/- and G5 *Tert*-/- mouse stained with H&E showing presence of many erythroid precursors in G0 *Tert*+/- and predominance of myeloid cells and relative erythroid hypoplasia in G5 *Tert*-/-.
