# Supplementary material for: Reversibility of Defective Hematopoiesis Caused by Telomere Shortening in Telomerase Knockout Mice
Source: PLoS One. 2015 Jul 2;10(7):e0131722. doi: 10.1371/journal.pone.0131722 (PMC4489842; doi:10.1371/journal.pone.0131722)
Supplement: S3 Fig — (DOCX) [file pone.0131722.s004.docx]

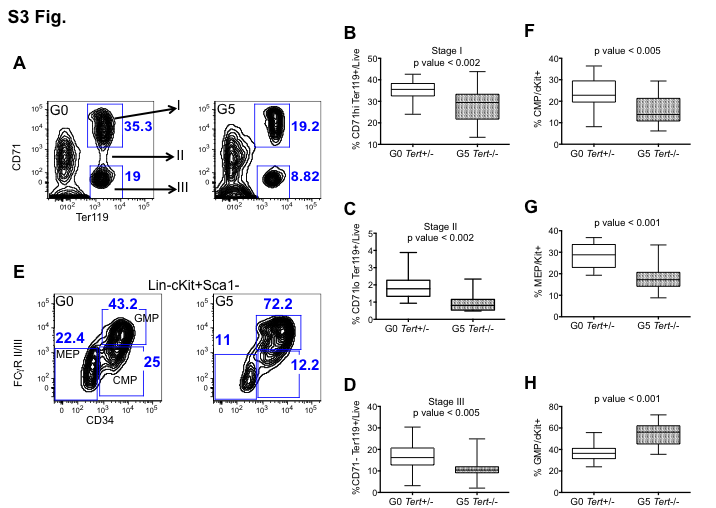


**S3 Fig. Defective Erythropoiesis in G5 *Tert*-/- mice.** (A) Representative flow cytometric (FACS) profiles of mouse BM pre-gated on live cells and separated based on levels of CD71 and Ter119 expression into three stages (I-III) of erythroid cell maturation. (B-D) Percentages of erythroblasts at stages I-III of erythroid maturation in G0 *Tert*+/- (n=21) and G5 *Tert*-/- (n=14) BM cells. (E) Representative FACS profiles of lineage-, c-Kit+ and Sca1- cells separated based on CD34 and FcγRII/III expression in G0 *Tert*+/- and G5 *Tert*-/- BM cells to define CMP (Lin-c-Kit+ Sca1- CD34+ FcγRII/IIIlo), MEP (Lin-c-Kit+ Sca1- CD34- FcγRII/III-) and GMP (Lin-c-Kit+ Sca1- CD34+ FcγRII/IIIhi) populations. (F-H) Percentages of CMP, MEP and GMP cells within the Lin-c-Kit+Sca1- population in G0 *Tert*+/- (n=21) and G5 *Tert*-/- (n=14) BM cells. The ends of the whiskers represent minimum and maximum values while the bar indicates the median value (50^th^ percentile). p values are based on a 2-tailed *t* test.
