# Supplementary material for: Reversibility of Defective Hematopoiesis Caused by Telomere Shortening in Telomerase Knockout Mice
Source: PLoS One. 2015 Jul 2;10(7):e0131722. doi: 10.1371/journal.pone.0131722 (PMC4489842; doi:10.1371/journal.pone.0131722)
Supplement: S4 Fig — (DOCX) [file pone.0131722.s005.docx]

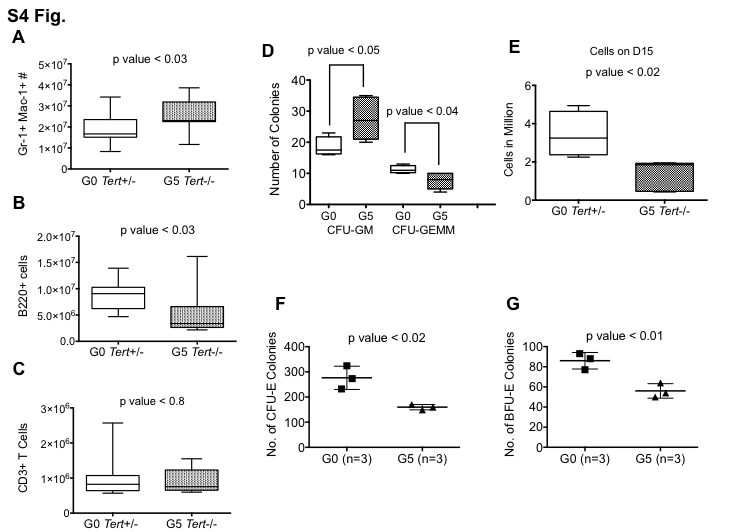


**S4 Fig. Leukocyte cell numbers and Colony Formation Assay from G0 *Tert*+/- and G5 *Tert*-/- Bone Marrow cells.**

(A) Myeloid cell (Gr1+Mac1+), (B) B cell (B220+) and (C) T cell (CD3+) numbers in the femurs from G0 *Tert*+/- (n=17) and G5 *Tert*-/- (n=11) mice aged 11-20 months. (D) Number of CFU-GM and CFU-GEMM colonies derived from 15,000 total BM cells from G0 *Tert*+/- (n=4) and G5 *Tert*-/- (n=5) mice. Cells were plated in Methocult M3434 that supports myeloid and erythroid colony formation. Colonies were counted 13-14 days after plating. (E) Total number of BM cells from G0 *Tert*+/- (n=4) and G5 *Tert*-/- (n=5) mice harvested on day 15 after culture in Methocult M3434. The ends of the whiskers represent minimum and maximum values while the bar indicates the median value (50^th^ percentile). p values are based on a 2-tailed *t* test. (F and G) Numbers of CFU-E and BFU-E colonies obtained from15,000 total BM cells from G0 *Tert*+/- (n=3) and G5 *Tert*-/- (n=3) mice after culture in Methocult M3334 that supports erythroid colony formation. CFU-E colonies were counted on day 3 and BFU-E colonies were counted on day 5 post-plating. Bars indicate standard deviation. p values are based on a 2-tailed *t* test.
