# Supplementary material for: Reversibility of Defective Hematopoiesis Caused by Telomere Shortening in Telomerase Knockout Mice
Source: PLoS One. 2015 Jul 2;10(7):e0131722. doi: 10.1371/journal.pone.0131722 (PMC4489842; doi:10.1371/journal.pone.0131722)
Supplement: S5 Fig — (DOCX) [file pone.0131722.s006.docx]

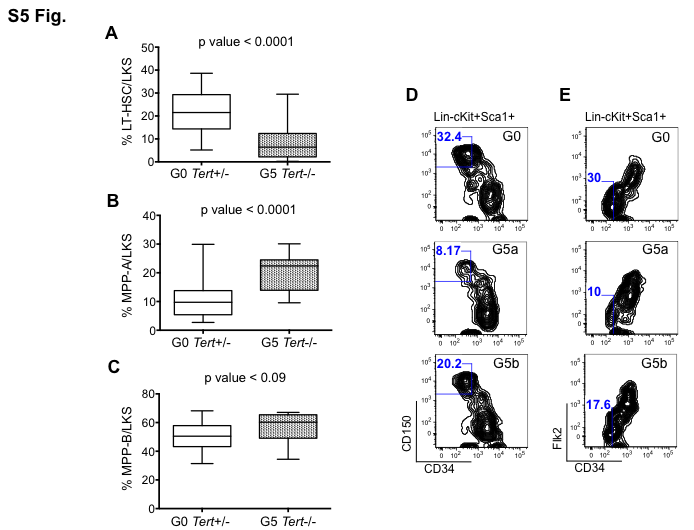


**S5 Fig. HSC and MPP cell populations in G5 *Tert*-/- mice.**

(A-C) Percentages of HSC, MPP-A and MPP-B cells within the Lin-c-Kit+Sca1+ population in the femurs from G0 *Tert*+/- (n=21) and G5 *Tert*-/- (n=14) mice aged 11-20 months. The ends of the whiskers represent minimum and maximum values while the bar indicates the median value (50^th^ percentile). p values are based on a 2-tailed *t* test. (D-E) Representative FACS profile pre-gated on live, lineage-, c-Kit+ and Sca1+ cells, separated based on either (D) CD34 and CD150 or (E) CD34 and Flk-2 expression showing reduced HSC frequency in G0 *Tert*+/- and G5 *Tert*-/- BM. G5a and G5b represent two different G5 mice, in total 3 mice of each genotype were studied.
