# Supplementary material for: Reversibility of Defective Hematopoiesis Caused by Telomere Shortening in Telomerase Knockout Mice
Source: PLoS One. 2015 Jul 2;10(7):e0131722. doi: 10.1371/journal.pone.0131722 (PMC4489842; doi:10.1371/journal.pone.0131722)
Supplement: S6 Fig — (DOCX) [file pone.0131722.s007.docx]

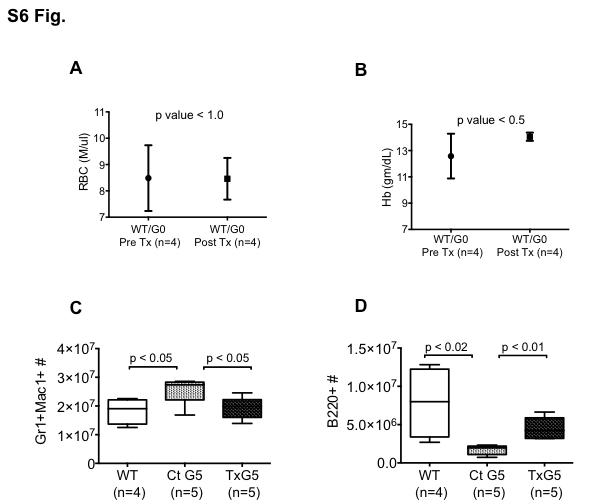


**S6 Fig. Tamoxifen Treated Control Mice and Myeloid Cell and B Cell numbers after Telomerase Reactivation**

(A and B) Comparison of RBC numbers and hemoglobin levels in the peripheral blood in tamoxifen treated wild type (WT) and G0 *Tert* +/- mice before and 3 months after treatment. (C and D) Number of myeloid (Gr-1+Mac1+) and B (B220+) cells in WT, G5 *Tert*-/- and tamoxifen treated G5 (TxG5) *Tert*-/- mice. Bars indicate standard deviation and p values are based on a 2-tailed *t* test.
