# Supplementary material for: Reversibility of Defective Hematopoiesis Caused by Telomere Shortening in Telomerase Knockout Mice
Source: PLoS One. 2015 Jul 2;10(7):e0131722. doi: 10.1371/journal.pone.0131722 (PMC4489842; doi:10.1371/journal.pone.0131722)
Supplement: S7 Fig — (DOCX) [file pone.0131722.s008.docx]

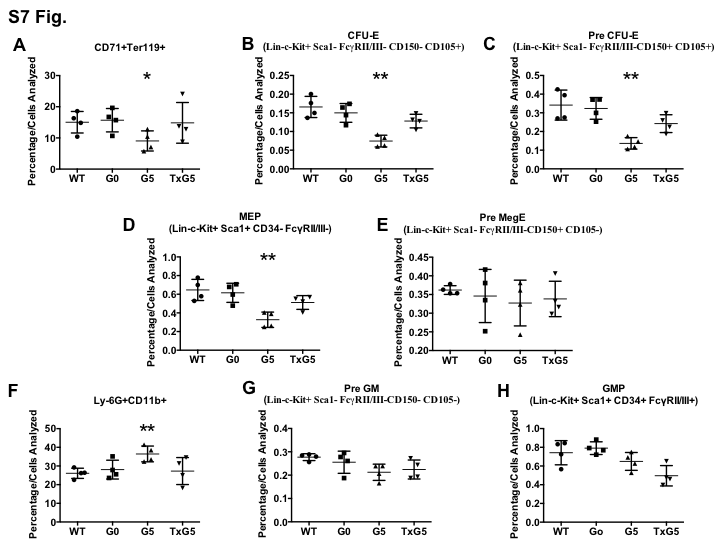


**S7 Fig. Changes in Cell Populations as Defined By Mass Cytometry.** Scatter plots showing percentages of (A) CD71+Ter119+ erythroblasts, (B) CFU-E, (C) Pre CFU-E, (D) MEP, (E) Pre MegE, (F) Myeloid, (G) Pre GM and (H) GMP populations in 11-14 months old WT *Tert*+/+ (n=4), G0 *Tert*+/- (n=4), G5 *Tert*-/- (n=4) and TxG5 *Tert*-/- (n=4) mice. Bars indicate standard deviation. p values are based on a 2-tailed *t* test. Statistically significant differences between WT and G5 *Tert*-/- mice are indicated by * (p value < 0.05) and ** (p value < 0.01). There were no significant differences between WT, G0 *Tert*+/- and TxG5 *Tert*-/- mice.
