# Supplementary material for: Reversibility of Defective Hematopoiesis Caused by Telomere Shortening in Telomerase Knockout Mice
Source: PLoS One. 2015 Jul 2;10(7):e0131722. doi: 10.1371/journal.pone.0131722 (PMC4489842; doi:10.1371/journal.pone.0131722)
Supplement: S8 Fig — (DOCX) [file pone.0131722.s009.docx]

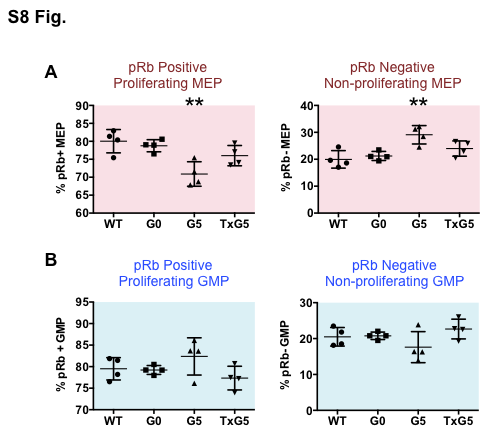


**S8 Fig. Cell Cycle Analysis in MEP, GMP and HSC Populations as Determined by Mass Cytometry.**

(A-B) Scatter plots showing percentage of proliferating pRb positive cells and non- proliferating pRb negative cells in (A) MEP and (B) GMP populations in WT *Tert*+/+ (n=4), G0 *Tert*+/- (n=4), G5 *Tert*-/- (n=4) and TxG5 *Tert*-/- (n=4) mice. Bars indicate standard deviation and the p values are based on a 2-tailed *t* test. Statistically significant differences between WT and G5 *Tert*-/- mice are indicated by ** (p value < 0.01). There were no significant differences between WT, G0 *Tert*+/- and TxG5 *Tert*-/- mice.
