# Supplementary material for: Reversibility of Defective Hematopoiesis Caused by Telomere Shortening in Telomerase Knockout Mice
Source: PLoS One. 2015 Jul 2;10(7):e0131722. doi: 10.1371/journal.pone.0131722 (PMC4489842; doi:10.1371/journal.pone.0131722)
Supplement: S9 Fig — (DOCX) [file pone.0131722.s010.docx]

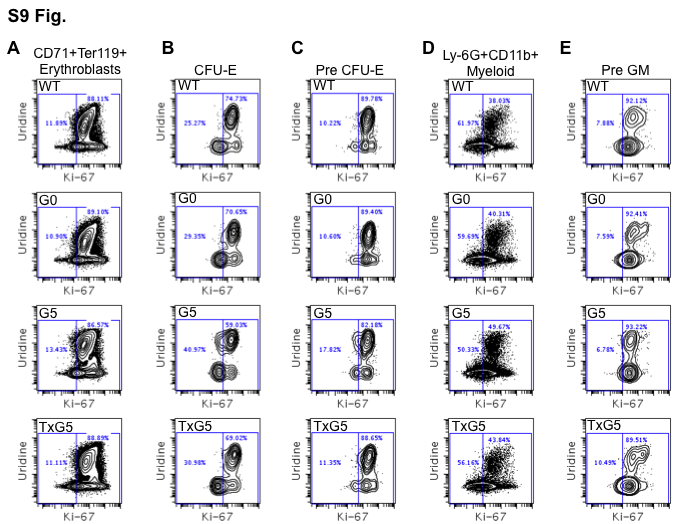


**S9 Fig. Cell Cycle analysis as Determined by Mass Cytometry in Different Erythoid and Myeloid populations.**

(A-E) Representative plots showing uridine incorporation and Ki-67 expression in (A) Erythroblasts (B) CFU-E, (C) Pre CFU-E, (D) mature myeloid cells and (E) pre GM populations in WT, G0 *Tert*+/-, G5 *Tert*-/- and TxG5 *Tert*-/- mice. Four mice of each genotype were studied.
