# Supplementary material for: Reversibility of Defective Hematopoiesis Caused by Telomere Shortening in Telomerase Knockout Mice
Source: PLoS One. 2015 Jul 2;10(7):e0131722. doi: 10.1371/journal.pone.0131722 (PMC4489842; doi:10.1371/journal.pone.0131722)
Supplement: S10 Fig — (DOCX) [file pone.0131722.s011.docx]

**S10 Fig.**

**B**

Ki-67+ MEP

Proliferating Cells

**A**

Non-Proliferating Cells

Ki-67- MEP

**S10 Fig. Apoptosis in MEP and GMP Populations as Determined by Mass Cytometry.**

(A-B) Scatter plots showing median cleaved PARP expression in (A) Ki-67+ proliferating and (B) Ki-67- non-proliferating cells in MEP populations in WT *Tert*+/+ (n=4), G0 *Tert*+/- (n=4), G5 *Tert*-/- (n=4) and TxG5 *Tert*-/- (n=4) mice. Bars indicate standard deviations and the p values are based on a 2-tailed *t* test. Negative values are the result of background subtraction of inter-event noise and the lack of background within cell events. Statistically significant difference between WT and G5 *Tert*-/- mice is indicated by ** (p value < 0.01). There were no significant differences between WT, G0 Tert+/- and TxG5 Tert-/- mice.
