# Supplementary material for: Reversibility of Defective Hematopoiesis Caused by Telomere Shortening in Telomerase Knockout Mice
Source: PLoS One. 2015 Jul 2;10(7):e0131722. doi: 10.1371/journal.pone.0131722 (PMC4489842; doi:10.1371/journal.pone.0131722)
Supplement: S1 Table — (DOCX) [file pone.0131722.s012.docx]

**S1 Table.** Sequence of primers used for genotyping mice.

| **Primers** | **Sequence 5' to 3'** |
| --- | --- |
| WT F | GCCAGCAATCAACTGACACTCG |
| WT R | CTCAGACGGTGCTCTGCAGC |
| Mutant R | CCAGCCTCTGTTCCACATACAGTAC |
| *Tert* in2F | GCCAGCAATCAACTGACACTCG |
| *Tert* in2R | ATGGGAACTTGGGAAGGAGAAGGG |
