# Supplementary material for: Reversibility of Defective Hematopoiesis Caused by Telomere Shortening in Telomerase Knockout Mice
Source: PLoS One. 2015 Jul 2;10(7):e0131722. doi: 10.1371/journal.pone.0131722 (PMC4489842; doi:10.1371/journal.pone.0131722)
Supplement: S2 Table — (DOCX) [file pone.0131722.s013.docx]

**S2 Table.** Antibodies used for mass cytometry analysis.

| **Antigen** | **Conjugate** | **Clone** | **Concentration** | **Manufacturer** |
| --- | --- | --- | --- | --- |
| **Surface Stain** |  |  |  |  |
| CD45 | In-115 | 30-F11 | 1.5ug/mL | Biolegend |
| Ly-6G | La-139 | 1A8 | 1ug/mL | Biolegend |
| CD71 | Nd-143 | R17217 | 2ug/mL | eBiosciences |
| CD44 | Nd-144 | IM7 | 2ug/mL | BD Biosciences |
| SlamF1 (CD150) | Nd-145 | TC15-12F12.2 | 1g/mL | Biolegend |
| CD4 | Sm-147 | RM4-5 | 1ug/mL | Biolegend |
| CD11b | Nd-148 | M1/70 | 0.25ug/mL | Biolegend |
| CD16/32 | Sm-149 | 93 | 2ug/mL | Biolegend |
| CD8 | Nd-150 | 53-6.7 | 1ug/mL | Biolegend |
| CD48 | Eu-151 | HM48-1 | 1.5ug/mL | eBiosciences |
| CD105 | Sm-154 | MJ7/18 | 3ug/mL | eBiosciences |
| CD41 | Gd-157 | MWReg30 | 2ug/mL | Biolegend |
| B220 | Gd-158 | RA3-6B2 | 1.5ug/mL | BD Biosciences |
| FLT3 | Gd-160 | A2F10 | 3ug/mL | eBiosciences |
| Ter119 | Dy-162 | TER-119 | 2ug/mL | Biolegend |
| cKit | Er-166 | ACK2 | 1ug/mL | eBiosciences |
| Sca-1 (Ly-6A/E) | Er-168 | E13-161.7 | 1ug/mL | Biolegend |
| CD3 complex | Yb-174 | 17A2 | 1.5ug/mL | BD Biosciences |
| CD34 | Lu-175 | RAM34 | 10ug/mL | BD Biosciences |
| **Intracellular Stain** |  |  |  |  |
| p16 | Pr-141 | G175-1239 | 3ug/mL | BD Biosciences |
| cleaved-Casp3 | Nd-142 | C92-605 | 2ug/mL | BD Biosciences |
| pATM (S1981) | Nd-146 | 10H11.E12 | 1.5ug/mL | Millipore |
| Ki-67 | Sm-152 | SolA15 | 1.5ug/mL | eBiosciences |
| pCHK2 (T68) | Eu-153 | Polyclonal | 3ug/mL | CST |
| Cyclin B1 | Gd-156 | GNS-1 | 3ug/mL | BD Biosciences |
| p53 | Dy-164 | 1C12 | 3ug/mL | CST |
| p-pRb (S807/811) | Ho-165 | J112-906 | 0.8ug/mL | BD Biosciences |
| pCDK1 (Y15) | Tm-169 | 10A11 | 4.5ug/mL | CST |
| pH2AX (S139) | Er-170 | JBW301 | 1ug/mL | Millipore |
| cleaved-PARP (Asp214) | Yb-171 | F21-852 | 2ug/mL | BD Biosciences |
| pRPS6 (S235/S236) | Yb-172 | N7-548 | 2ug/mL | BD Biosciences |
| pHistone H3 (S28) | Yb-176 | HTA28 | 0.5ug/mL | Bioloegend |
